# Ultrasound-Guided Erector Spinae Plane Block in Thoracolumbar Spinal Surgery: A Systematic Review and Meta-Analysis

**DOI:** 10.3389/fmed.2022.932101

**Published:** 2022-07-04

**Authors:** Dmitriy Viderman, Mina Aubakirova, Yerlan Umbetzhanov, Gulnara Kulkaeva, S. B. Shalekenov, Yerkin G. Abdildin

**Affiliations:** ^1^Nazarbayev University School of Medicine (NUSOM), Nur-Sultan, Kazakhstan; ^2^National Research Oncology Center, Nur-Sultan, Kazakhstan; ^3^Nazarbayev University School of Engineering and Digital Sciences, Nur-Sultan, Kazakhstan

**Keywords:** regional anesthesia, erector spinae plane block, spinal surgery, pain management, post-operative analgesia, opioid consumption

## Abstract

**Introduction:**

Neurosurgical spinal surgeries such as micro- discectomy and complex fusion surgeries remain the leading causes of disability-adjusted life-year. Major spinal surgeries often result in severe postprocedural pain due to massive dissection of the underlying tissues. While opioids offer effective pain control, they frequently lead to side effects, such as post-operative nausea and vomiting, pruritus, constipation, and respiratory depression. ESPB was successfully used in spinal surgery as a component of a multimodal analgesic regimen and it eliminated the requirements for opioids. The primary purpose of this systematic review and meta-analysis was to compare post-operative opioid consumption between ESPB and placebo.

**Methods:**

To conduct this systematic review, we used the “Preferred Reporting Items for Systematic Reviews and Meta-Analyses (PRISMA)” guidelines. We conducted a search for relevant articles available in the following databases: Google Scholar, PubMed, and the Cochrane Library published up to March 2022.

**Results:**

The total morphine consumption within 24 h after surgery was lower in the ESPB group, the mean difference (in mg of morphine) with 95% CI is −9.27 (−11.63, −6.91). The pain intensity (0–10) at rest measured 24 h after surgery was lower in the ESPB group, the MD with 95% CI is −0.47 (−0.77, −0.17). The pain intensity during movement measured 24 h after surgery was lower in the ESPB group, the MD with 95% CI is −0.73 (−1.00, −0.47). Post-operative nausea and vomiting were significantly lower in the ESPB group, the risk ratio with 95% CI is 0.32 (0.19, 0.53).

**Conclusion:**

Ultrasound-guided ESPB was superior to placebo in reducing post-operative opioid consumption, pain intensity, post-operative nausea and vomiting, and prolonging the time to first rescue analgesia. There were no ESPB-related serious complications reported.

## Introduction

Neurosurgical spinal surgeries comprise procedures ranging from micro-discectomy to complex fusion surgeries remain highly prevalent (1). This prevalence of these procedures is driven by the “epidemy” of low back pain, which is one of the leading causes of disability-adjusted life-years (2, 3). Major spinal surgeries are often associated with severe postprocedural pain due to massive dissection of the underlying tissues (the skin, subcutaneous tissue, ligaments, and osseous structures) (3). Opioids are one of the commonly used analgesics for perioperative management of acute pain after spinal neurosurgical procedures (3). While opioids offer effective pain control, they frequently lead to side effects, such as post-operative nausea and vomiting, pruritus, constipation, and respiratory depression (4). To minimize or eliminate these side effects, interfascial plane blocks are increasingly used to improve the quality of post-operative pain management (5).

Thus ESPB has demonstrated its efficacy in the management of various acute and chronic pain-related conditions (6–13).

ESPB block acts on the posterior rami of spinal nerves (4). Local anesthetics injected in the erector spinae plane spreading over the paravertebral and epidural spaces block the posterior rami of spinal nerves, the anterior and posterior rami of the spinal nerves (14). ESPB was first used in spinal surgery as a component of a multimodal analgesic regimen and it eliminated the requirements for opioids (15).

The primary purpose of this systematic review and meta-analysis was to compare post-operative opioid consumption between ESPB and control groups. The secondary purposes were to evaluate the efficacy of ESPB in acute pain management, the time to the first opioid requirement, and the frequency of post-operative nausea and vomiting.

## Materials and Methods

### Protocol

To prepare this systematic review, we followed the “Preferred Reporting Items for Systematic Reviews and Meta-Analyses (PRISMA)” (16).

We created a protocol of the systematic review with the inclusion and exclusion criteria for relevant articles. The protocol and methods of analysis were approved by all authors (Supplementary File 1). We searched for randomized controlled trials (RCTs) that compared the analgesic effects of ESPB with control.

#### Inclusion Criteria

1) Randomized controlled trials (RCT);
2) 18 years and older;
3) Studies comparing ESPB (bilateral single shot) and control in spinal surgery;
4) Pain management methods assessed using the standard scales, VAS (visual analog scale) or NRS (numerical rating scale) were considered.

#### Exclusion Criteria

1) Observational studies, case reports or series, editorials, cadaver studies, technical reports;
2) Not detailed description of methodology, outcomes, results.

#### PICO Criteria

We selected studies that met the following criteria:

Population: 18 years and older undergoing thoracolumbar spinal surgeries;

Intervention: erector spinae plane block;

Comparator: control - placebo (sham);

Outcomes: primary – opioid consumption during the first 24 h after surgery;

Secondary – pain scores after surgery; time to first rescue opioid request; the presence of side effects of opioids (e.g., nausea, vomiting, respiratory depression, pruritis); side effects and complications such as mechanical injury by the needle, local anesthetic systemic toxicity (LAST).

Studies to be considered for inclusion: randomized controlled clinical trials.

### Search Methods

We conducted a search for relevant articles available in the following databases: PubMed, the Cochrane Library, and Google Scholar, published during the period from inception to March 2022. The search included the following search terms or their combination ((((“erector spinae plane block,”) “erector spinae block,”) “ESP block,”) “ESPB”) AND (((“spinal surgery,”) “lumbar spine surgery”) OR “spine surgery”) (Supplementary File 2).

### Data Extraction and Statistical Methods

We entered data in a data table. The following information was included: reference, 1-st author, year of publication, types of surgery, sample size, time of the block, adverse events, and complications.

We recalculated the data given in a median and interquartile range, the mean, and standard deviation using the approach developed by Luo et al. (17) for the sample mean and by Wan et al. (18) for sample standard deviation. To standardize outcome measures, we converted post-operative opioid doses into intravenous morphine equivalents (mg) (19, 20).

To convert sufentanil (mcg), fentanyl (mcg), tramadol (mg), oxycodone (mg), and pethidine (mg) consumption into morphine (mg) consumption we used the following multiplicators: 0.5, 0.1, 0.1, 1.5, and 0.1, respectively. We utilized Review Manager 5.4.1 for constructing the forest plots.

Data analysis was conducted using the “Review Manager software (RevMan, version 5.4).” Statistical heterogeneity was estimated by the I^2^ statistic.

### Assessment of Methodological Quality

We evaluated the methodological quality of the included studies using the “Cochrane risk of bias assessment” scale and Jadad scale.

## Results

**FIGURE 1:**
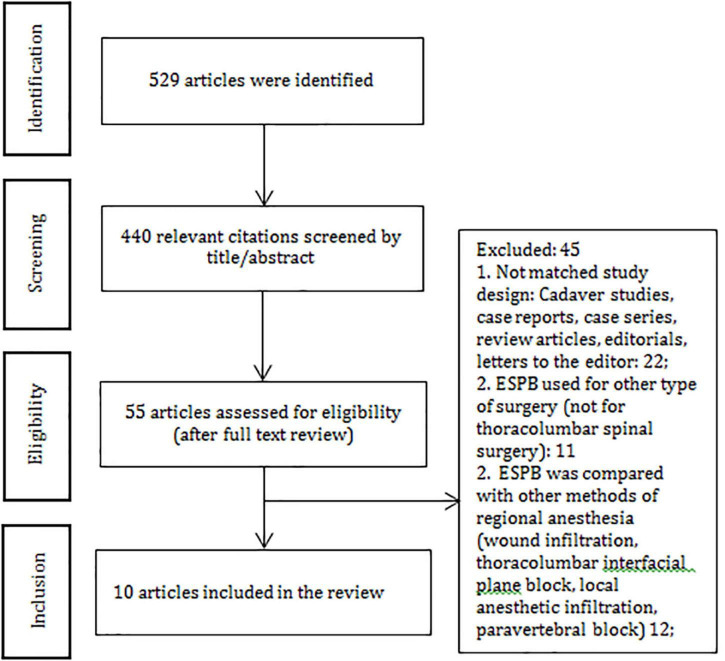
PRISMA diagram.

In total, 529 articles were initially identified through a systematic search. Fifty-five articles were assessed for eligibility, 45 articles did not match the criteria and were excluded. Ten articles were included in the systematic review and analyzed (Figure 1 and Supplementary File 2). We extracted the data related to post-operative opioid consumption, the efficacy of ESPB in pain relief, timing to the first opioid requirement, the rate of post-operative side-effects and complications in the ESPB group and control group. Six hundred fifty-one patients (ESPB group – 327 and control group – 324) aged 18–80 were included (Table 1).

**TABLE 1:** Characteristics of included studies.

Author, citation	Country	Study design	Study goals	Age	Number of patients	Surgery	General anesthesia	ASA	Levels of ESPB	LA
Ciftci et al. (14)	Turkey	RCT	Primary- postop. opioid consumption. Secondary- to compare the post-operative pain scores, the use of rescue analgesia, the block procedure times, and the adverse effects of opioids	18–65	60 (30/30)	Spinal fusion surgery	Yes	I–II	L3	20 mL of 0.25% bupivacaine bilaterally (40 ml bilaterally)
Finnerty (27)	Ireland	RCT	Primary-summed QoR-15 score at 24 post-operative hours, secondary- pain intensity and opioid Consumption, time to first intravenous opioid demand	59	60 (30/30)	Thoraco-lumbar spinal surgery	Yes	I–IV		40 ml levobupivacaine 0.25% bilaterally
Goel (25)	India	RCT	Total opioid consumption Total muscle relaxant consumption Total intraoperative blood loss (ml) Total satisfaction score	18 –78	100 (50/50)	Transforaminal Lumbar Inter-body Fusion surgery	Yes	I–II		20 ml of 0.25% bupivacaine (40 ml bilaterally)
Singh (22)	India	RCT	Primary- a 24-h cumulative morphine consumption 24 h after surgery. Secondary pain intensity, patient satisfaction score	18 –65	40 (20/20)	Lumbar spine surgery (lumbar stenosis, prolapsed lumbar intervertebral disk, Laminectomy)	Yes	I–II	C7-T10;	20 mL of 0.5% Bupivacaine (40 ml bilaterally)
Yayik et al. (26)	Turkey	RCT	To measure postop tramadol consumption	18–65	60 (30/30)	Open lumbar decompression surgery	Yes	I–III	L3	0.25% bupivacaine 20 mL (40 ml bilaterally)
Yesiltas 2021 (23)	Turkey	RCT	Efficacy of ESPB on pain scores		56 (28/28)	Spinal fusions for spondylolisthesis	Yes	I–III		20 mL (1:1) 0.25% bupivacaine and 1.0% lidocaine
Yörükoğu (21)	Turkey	RCT (double blind)	Primary-morphine consumption (24 h); Secondary-morphine consumption at 1st, 6th and 12 th, pain intensity, PONV	18–65	54 (28 ESPB/26 control)	Lumbar disk hernia surgery	Yes	I–II	L4	20 mL of 0.25% bupivacaine (40 ml bilaterally)
Yu (24)	China	RCT	Pain intensity	26–67	80 (40/40)	Dorsal lumbar spinal surgery due to lumbar spinal fractures	Yes	I–III	T7	30 mL of 0.25% bupivacaine (60 ml bilaterally)
Zhang et al. (15)	China	RCT (blinded)	Pain intensity; post-operative sufentanil consumption; sufentanil requirement after surgery Adverse effects; recovery	20–75	60 (30/30)	Spinal fusion surgery	Yes	I–III	L3 or L4	20 mL 0.4% ropivacaine was injected (40 ml bilaterally)
Zhu et al. (39)	China	RCT	Primary - dosage of oxytocin, secondary – remifentanil consumption, adverse effects, pain scores, hypoesthesia range	45–70	40 (20/20)	Lumbar fusion	Yes	I–II	L2	ropivacaine 0.375% (20 mL, bilaterally)

Spinal neurosurgical procedures included spinal fusion surgery, lumbar stenosis, prolapsed lumbar intervertebral disk, laminectomy, open lumbar decompression surgery, lumbar disk hernia surgery, dorsal lumbar spinal surgery spinal fusion surgery. All patients enrolled in the studies received general anesthesia apart from ESPB. Only patients of the American society of anesthesiologists’ status I–II were considered for inclusion in the studies. ESPB was performed at the level from T10 to L4 (Table 1).

The following local anesthetics were used in the RCTs: bupivacaine (in seven studies), ropivacaine (in one study), levobupivacaine (in one study), and a combination of bupivacaine and lidocaine (in one study). The authors used the volume of LA ranging from 20 to 30 ml (40 and 60 ml bilaterally), the concentration of ropivacaine of 0.4% and the concentration of bupivacaine ranging from 0.25 to 0.5%. Geographically, five out of ten RCTs were conducted in Turkey, two in China, two – in India, and one – in Ireland (Table 1). All reported that ultrasound-guided ESPB was superior to placebo in reducing post-operative opioid consumption, pain intensity scores, post-operative nausea and vomiting (PONV), and extending the time to the first rescue analgesia demand. There were no reports on serious complications related to ESPB.

### Total Opioid Consumption Within 24 h After Surgery

The authors used different types of opioids and their concentrations in the post-operative period. Thus, Yörükoğlu et al. (21), Singh et al. (22), and Yeşiltaş et al. (23) reported the total morphine consumption in mg, Yu et al. (24) and Zhang et al. (15) reported cumulative sufentanil consumption in mg and μg (respectively), Cifci et al. (14) and Goel et al. (25) – fentanyl consumption in mcg, Yaiyk et al. (26) – tramadol in mg, Finnerty et al. (27) – oxycodone in mg, and Eskin et al. (28) – the total PCA pethidine dose in mg.

**FIGURE 2:**
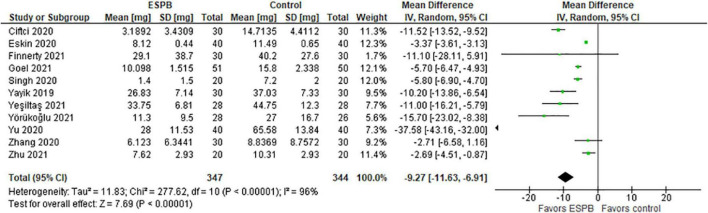
Total opioid consumption within 24 h after surgery in mg of morphine.

The total morphine consumption within 24 h after surgery is presented in a forest plot (Figure 2). The model favors ESPB over control because the total opioid consumption within 24 h after surgery was considerably lower in the ESPB group compared with the control, the mean difference with 95% confidence interval (CI) is −9.27 (−11.63, −6.91). Due to the different populations in the studies, we constructed the model with the random-effects analysis. The total number of patients in the ESPB groups is 347, while in the control group there are 344 patients. According to the studies, the patients were randomly assigned to these groups by means of a computer program, and the nurses were blinded to the patients’ assignment to the groups. The value of I^2^ is equal to 96%, so the model shows high heterogeneity and this is significant since the *p*-value < 0.00001. Due to the high heterogeneity of the studies, we performed the sensitivity analysis by excluding one study at a time, but this did not significantly affect the overall result, the model still favors ESPB over control.

### Pain Intensity (NRS/VAS) Scores at Rest Recorded 24 h After Surgery

**FIGURE 3:**
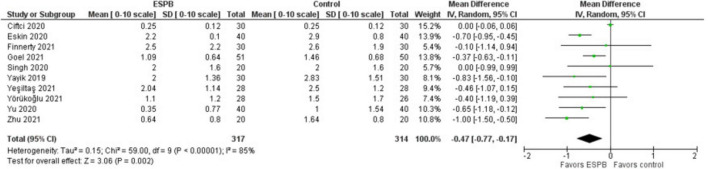
Pain intensity at rest measured 24 h after surgery.

The pain intensity at rest measured 24 h after surgery is presented in a forest plot (Figure 3). It shows that the patients were more satisfied after the surgery when the ESPB was applied compared to the patients in the control group, the mean difference with 95% CI is −0.47 (−0.77, −0.17). This result is insensitive to the exclusion of any study. Zhang et al. (15) provided data values in graphical format only, so we were unable to use their results in this analysis. Some studies reported patient satisfaction in either NRS or VAS at different hours after surgery for both settings: at rest (or, passive) and during movement (or, active, while mobilized). Finnerty et al. (27) used an “11-point verbal response scale” without mentioning NRS and VAS.

### Pain Intensity (NRS/VAS) Scores During Movement Measured 24 h After Surgery

**FIGURE 4:**
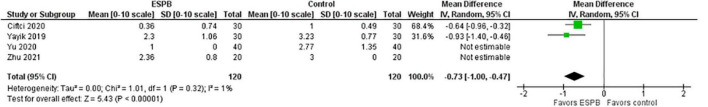
Pain intensity during movement measured 24 h after surgery.

The pain intensity measured as NRS/VAS score during movement measured 24 h after surgery is presented in a forest plot (Figure 4). It shows that the patients were more satisfied after the surgery when the ESPB was applied compared to the patients in the control group, the mean difference with 95% CI is −0.73 (−1.00, −0.47). This result is insensitive to the exclusion of any study.

### Post-operative Nausea and Vomiting

**FIGURE 5:**
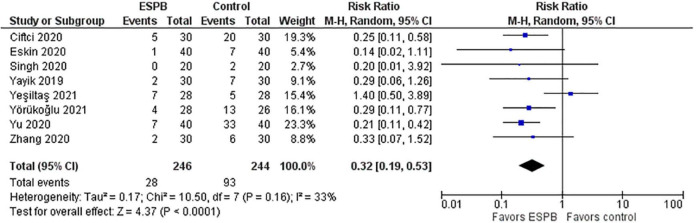
Post-operative nausea and vomiting.

Post-operative nausea and vomiting (PONV) in the ESPB and control groups are depicted in a forest plot (Figure 5). The analysis favors ESPB over control because the number of patients with PONV in the ESPB groups was significantly lower than those in the control groups; the risk ratio with 95% CI is 0.32 (0.19, 0.53). The result is insensitive to the exclusion of any study.

### Effect of ESPB on Rescue Analgesia

**FIGURE 6:**
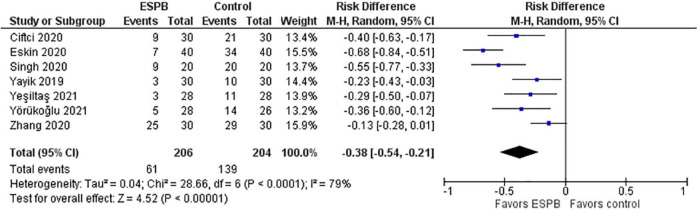
The number of patients requiring rescue analgesia after surgery.

The effect of the ESPB on the necessity to use rescue analgesia is presented in a forest plot (Figure 6). The number of patients who required rescue analgesia after surgery was considerably lower in the ESPB groups than in the control groups. The studies utilized different opioids for rescue analgesia. In particular, Ciftci et al. (14) reported the use of meperidine as rescue analgesia, Singh et al. (22) – morphine, Eskin et al. (28), Yayik et al. (26), and Yeşiltaş et al. (23) – pethidine, Yörükoğlu et al. (21) – tenoxicam 20 mg intravenously, and Zhang et al. (15) – sufentanil.

### Methodological Quality of Included Studies

The methodological quality of the studies is shown in Table 2 (Cochrane risk of bias scale) and Supplementary File 3 (Jadad scale).

**TABLE 2:** Cochrane risk-of-bias.

	Randomization bias (selection bias)	Allocation concealment (selection bias)	Blinding of participants and personnel (performance bias)	Blinding of outcome assessment (detection bias)	Incomplete outcome data (attrition bias)	Selective reporting (reporting bias)	Other bias
Yörükoğlu et al. (21)	“+”	“+”	“+”	“+”	“+”	“–”	?
Yu et al. (24)	“+”	“–”	“–”	“–”	“+”	“+”	?
Zhang et al. (15)	“+”	“+”	“+”	?	“+”	“+”	?
Ciftci et al. (14)	“–”	“–”	“–”	?	“–”	“+”	?
Singh et al. (22)	“+”	“–”	“–”	“–”	“+”	“+”	?
Yayik et al. (26)	“+”	“+”	“–”	“+”	“+”	“–”	?
Goel et al. (25)	“+”	“–”	“+”	“+”	“–”	“–”	?
Finnerty et al. (27)	“+”	“–”	?	“–”	“–”	“–”	?
Yeşiltaş et al. (23)	“+”	“+”	“+”	“+”	“+”	“–”	?
Zhu et al. (39)	“+”	“+”	“–”	“+”	“+”	?	?

*“+” – low risk of Bias (green).*

*“–” – high risk of bias (red).*

*? – undetermined (yellow).*

## Discussion

Current systematic review and meta-analysis present evidence on the clinical role of ESPB in pain management after spinal surgery. ESPB was found to reduce the cumulative opioid consumption within 24 h after surgery, reduce pain severity (NRS/VAS) scores at rest measured 24 h after surgery, post-operative side-effects such as nausea and vomiting, and reduce the number of patients requiring rescue analgesia after surgery.

Patients after spinal surgeries frequently may complain of moderate-to-severe pain and post-operative analgesia is essential for early mobilization and overall satisfaction (16, 29). Moreover, adequate pain management is also an important measure to prevent post-operative atelectasis, deep vein thrombosis and thromboembolism (16, 29).

Post-operative pain in lumbar spinal surgery originates from surgical retraction and mechanical injury, and denervation of bone, muscles, ligaments, zygapophysial joints, intervertebral discs innervated by the dorsal rami of spinal nerves.

The mechanism of pain is multi-factorial and combining nociceptive, neuronal, and inflammatory components; therefore, patient-controlled intravenous opioid analgesia might be insufficient (25). Furthermore, opioid-related side effects such as nausea or vomiting, hypoventilation hypotension, in severe cases – respiratory depression, or loss of consciousness limit the use of opioids in the post-operative period (30). ESPB offers a multidermatomal sensory block through the blockage of the posterior and anterior (not consistently blocked) rami of the thoracic spinal nerves; moreover, the craniocaudal spread of local anesthetics enhances its analgesic efficacy (31, 32).

Although ESPB is considered an interfascial block paraspinal block, one of the components of its analgesic efficacy is explained by the spread of LAs to the paravertebral and epidural spaces (31, 33). The ESPB blocks both parietal and visceral sensations. One of the hypothetical mechanisms of ESPB is the spread of local anesthetics in paravertebral space reaching the ventral and dorsal rami (dorsal rami are always involved) of the spinal nerves as well as the communicating branches of the sympathetic chain (34, 35). Therefore, this effect resembles the paravertebral block. The local anesthetic covers a wide area through caudal and cephalic diffusion. Moreover, if ESPB is performed at the lumbar region, high volumes of local anesthetics might diffuse to the lumbar plexus (35). Finally, another potential mechanism of ESPB is systemic absorption of local anesthetics (34).

One of the most important explanations for the popularity of ESPB is its simplicity in sonographic identification of anatomical landmarks and a better safety profile in comparison with paravertebral block (26).

Benefits of the ESPB include the simplicity of performance with precise ultrasound-guided anatomic. Additionally, ESPB is safe; the injection site is distant to the major vessels and nerves. Therefore, the risk of intravascular administration of local anesthetics or nerve injury due to neuroaxial puncture is low.

Pain scores were lower immediately after surgery and during the early post-operative period in patients that received ESPB. The ESPB reduced the dose of opioids required in the post-operative period and improved patient satisfaction. There were no complications related to the ESPB reported.

There were no significant differences in intraoperative outcomes such as intraoperative opioid dose, episodes of hypotension, duration of surgery, and blood loss. Early post-operative outcomes include time to extubation, length of ICU stay, ambulation time, surgical complications, and hospital length of stay.

Zhang et al. (15) found that the highest difference in post-operative NRS was during the first 8 h after surgery. After 8 h following surgery, the difference was minimal (15). Although the analgesic effects of ESP block lasted at rest for 12 h after surgery, there was no significant difference in pain scores between the two groups on movement beyond 4 h.

One of the explanations for the limited pain relief during movement is that the local anesthetic distribution varies with patient position, pressure on the compartment by muscle tone, and anatomical variation, therefore, the area of sensory loss after ESPB might not cover the multi-level incision area (15).

ESPB has some advantages over other types of blocks used for analgesia in spinal surgeries, such as thoracolumbar interfascial plane block (TLIP block) (14) that is used for minor spinal surgeries. TLIP block is performed by injecting the LA into the fascial plane within the erector spinae muscle. Although procedure seems to be simple, it might be challenging in patients undergoing revision spine surgeries and obese patients due to difficulties in identifying muscles in such individuals (14). Retrolaminar block is similar to ESPB and TLIP block and performed by injecting the LA deep into the erector spinae muscle, but the anatomic target is the lamina. ESP block results in a wider spread of LA laterally and craniocaudally if performed at the T5 vertebral level (36). The addition of ESPB to multimodal analgesia after thoracolumbar decompressive spinal surgery improved recovery and reduced post-operative pain. ESPB added to multimodal analgesia might improve the outcomes in enhanced recovery after surgery (25).

### Side Effects and Complications of ESPB

We did not find any information regarding the side effects and complications of ESPB in the included studies. ESPB has been shown to have low risks of hypocoagulation-related complications, which might be a limiting factor for epidural anesthesia. There are no major vessels located close to the place of needle placement; the risks of hematoma formation and local anesthetic systemic toxicity due to intravascular injections are lower compared with other regional anesthetic blocks (37). Nonetheless, despite no major complications reported, the detection and management of complications, such as LAST should be recognized (38).

## Limitations

The main limitations of this systematic review are small sample sizes, single centered studies with tight inclusion and exclusion criteria that might not be representative of a real-world picture or patients in other medical centers. One study mentioned that anesthesiologists, surgeons, investigators, and patients were not blinded to the intervention. Therefore, it might have added an element of bias. Finally, high values of I^2^ > 60 (Figures 2– 5) suggest a high level of heterogeneity.

## Conclusion

This meta-analysis showed that ultrasound-guided ESPB was superior to placebo in reducing post-operative opioid consumption, pain intensity, post-operative nausea and vomiting, and prolonging the time to first rescue analgesia. There were no ESPB-related serious complications reported.

## Data Availability Statement

Inquires can be directed to corresponding author.

## Author Contributions

DV: conceptualization, writing—original draft preparation, and funding acquisition. DV and YA: methodology, writing—review and editing, and project administration. YA: software, formal analysis, and visualization. MA, YU, GK, and SS: data curation. All authors have read and agreed to the published version of the manuscript.

## Conflict of Interest

The authors declare that the research was conducted in the absence of any commercial or financial relationships that could be construed as a potential conflict of interest.

## Publisher’s Note

All claims expressed in this article are solely those of the authors and do not necessarily represent those of their affiliated organizations, or those of the publisher, the editors and the reviewers. Any product that may be evaluated in this article, or claim that may be made by its manufacturer, is not guaranteed or endorsed by the publisher.
