# Supplementary material for: Ultrasound-Guided Erector Spinae Plane Block in Thoracolumbar Spinal Surgery: A Systematic Review and Meta-Analysis
Source: Front Med (Lausanne). 2022 Jul 4;9:932101. doi: 10.3389/fmed.2022.932101 (PMC9289466; doi:10.3389/fmed.2022.932101)
Supplement: Supplementary file 1 [file Data_Sheet_1.docx]

**Supplementary file 1.**

**Protocol of systematic review and meta-analysis.**

**Ultrasound-guided Erector Spinae Plane Block in thoracolumbar Spinal Surgery: A systematic review and meta-analysis**

We designed a methodology and protocol for the systematic review with the inclusion and exclusion criteria for relevant articles. The protocol and methods of analysis were approved by all authors. To prepare this systematic review, we will follow the “Preferred Reporting Items for Systematic Reviews and Meta-Analyses (PRISMA)”. We will search randomized controlled trials (RCTs) that compared the analgesic effects of ESPB with “no block”.

**Inclusion criteria:**

1) Randomized controlled trials (RCT);

2) 18 years and older;

3) Studies comparing ESPB (bilateral single shot) in spinal surgery and no block pain management methods assessed using the standard scales, VAS (visual analog pain score) or NRS (numerical pain rating score) were considered;

**Exclusion criteria:**

1) Non-RCTs: case reports or series, editorials, cadaver studies, retrospective studies, technical reports;

2) Not detailed description of methodology, outcomes, results

**PICO criteria:**

We will select studies that meet the following criteria:

Population: 18 years and older undergoing thoracolumbar spinal surgeries;

Intervention: erector spinae plane block

Comparator: No block or placebo (sham);

Outcomes: Primary – opioid consumption during the first 24 hours after surgery;

Secondary – pain scores after surgery; time to first rescue opioid request; the presence of side effects of opioids (e.g., nausea, vomiting, respiratory depression, pruritis); side effects and complications such as mechanical injury by the needle, local anesthetic systemic toxicity (LAST).

**Studies to be considered for inclusion**: randomized controlled clinical trials.

**Search methods**

We will conduct a search for relevant articles available in the following databases: Google Scholar, PubMed, and the Cochrane Library published during the period from the inception to March 2022. The search will include the following search terms and/or their combination ((((“erector spinae plane block,”) “erector spinae block,”) “ESP block,”) “ESPB”) AND (((“spinal surgery,”) “lumbar spine surgery”) OR “spine surgery”):

((erector spinae plane block) AND (spinal surgery)) Sort by: Most Recent

("erector"[All Fields] OR "erectores"[All Fields] OR "erectors"[All Fields]) AND "spinae"[All Fields] AND ("aircraft"[MeSH Terms] OR "aircraft"[All Fields] OR "plane"[All Fields] OR "planes"[All Fields]) AND ("block"[All Fields] OR "blocked"[All Fields] OR "blocking"[All Fields] OR "blockings"[All Fields] OR "blocks"[All Fields]) AND (("spinal"[All Fields] OR "spinalization"[All Fields] OR "spinalized"[All Fields] OR "spinally"[All Fields] OR "spinals"[All Fields]) AND ("surgery"[MeSH Subheading] OR "surgery"[All Fields] OR "surgical procedures, operative"[MeSH Terms] OR ("surgical"[All Fields] AND "procedures"[All Fields] AND "operative"[All Fields]) OR "operative surgical procedures"[All Fields] OR "general surgery"[MeSH Terms] OR ("general"[All Fields] AND "surgery"[All Fields]) OR "general surgery"[All Fields] OR "surgery s"[All Fields] OR "surgerys"[All Fields] OR "surgeries"[All Fields]))

**Data extraction and statistical methods**

We will enter data in a data table. The following information will be included: reference, 1-st author, year of publication, types of surgery, sample size, time of the block, adverse events, and complications.

We will recalculate the data given in a median and interquartile range, the mean, and standard deviation using the approach developed by Luo et al. for the sample mean and by Wan et al. [12] for the sample standard deviation. To standardize outcome measures, we will convert postoperative opioid doses into intravenous morphine equivalents (mg).

To convert sufentanil (mcg), fentanyl (mcg), tramadol (mg), oxycodone (mg), and pethidine (mg) consumption into morphine (mg) consumption we will use the following multiplicators: 0.5, 0.1, 0.1, 1.5, and 0.1, respectively. If studies report only median values and the interquartile ranges (or minimum, median, and maximum values, we will utilize the existing estimation techniques to calculate the sample mean and the sample standard deviation. We will utilize Review Manager 5.4.1 for constructing the forest plots.

Data analysis will be conducted using the “Review Manager software (RevMan, version 5.4)”. Statistical heterogeneity will be estimated by the I^2^ statistic.

**Assessment of methodological quality**

We will evaluate the methodological quality of the included studies using the Oxford quality scoring system (Jadad Scale). The methodological quality of studies was graded on a scale from 1 (min) to 5 (max).
