# Supplementary material for: Ultrasound-Guided Erector Spinae Plane Block in Thoracolumbar Spinal Surgery: A Systematic Review and Meta-Analysis
Source: Front Med (Lausanne). 2022 Jul 4;9:932101. doi: 10.3389/fmed.2022.932101 (PMC9289466; doi:10.3389/fmed.2022.932101)
Supplement: Supplementary file 2 [file Data_Sheet_2.docx]

***Supplementary file 2.***

***Search strategy for the identification of articles.***

*(("erector"[All Fields] OR "erectores"[All Fields] OR "erectors"[All Fields]) AND "spinae"[All Fields] AND ("aircraft"[MeSH Terms] OR "aircraft"[All Fields] OR "plane"[All Fields] OR "planes"[All Fields]) AND ("block"[All Fields] OR "blocked"[All Fields] OR "blocking"[All Fields] OR "blockings"[All Fields] OR "blocks"[All Fields]) AND (("lumbar vertebrae"[MeSH Terms] OR ("lumbar"[All Fields] AND "vertebrae"[All Fields]) OR "lumbar vertebrae"[All Fields] OR ("lumbar"[All Fields] AND "spine"[All Fields]) OR "lumbar spine"[All Fields]) AND ("surgery"[MeSH Subheading] OR "surgery"[All Fields] OR "surgical procedures, operative"[MeSH Terms] OR ("surgical"[All Fields] AND "procedures"[All Fields] AND "operative"[All Fields]) OR "operative surgical procedures"[All Fields] OR "general surgery"[MeSH Terms] OR ("general"[All Fields] AND "surgery"[All Fields]) OR "general surgery"[All Fields] OR "surgery s"[All Fields] OR "surgerys"[All Fields] OR "surgeries"[All Fields]))) OR (("spine"[MeSH Terms] OR "spine"[All Fields] OR "spines"[All Fields] OR "spine s"[All Fields]) AND ("surgery"[MeSH Subheading] OR "surgery"[All Fields] OR "surgical procedures, operative"[MeSH Terms] OR ("surgical"[All Fields] AND "procedures"[All Fields] AND "operative"[All Fields]) OR "operative surgical procedures"[All Fields] OR "general surgery"[MeSH Terms] OR ("general"[All Fields] AND "surgery"[All Fields]) OR "general surgery"[All Fields] OR "surgery s"[All Fields] OR "surgerys"[All Fields] OR "surgeries"[All Fields]))*

*529 articles were identified: PubMed- 140, Cochrane – 14; Google Scholar -375*

*440 relevant citations screened by title and abstract*

*55 articles assessed for eligibility*

*Excluded: 45*

*1) Not matched study design: Cadaver studies, case reports, case series, review articles, editorials, letters to the editor: 22;*

*2) ESPB used for other type of surgery (not for thoracolumbar spinal surgery): 11*

*3) ESPB was compared with other methods of regional anesthesia (wound infiltration, thoracolumbar interfacial plane block, local anesthetic infiltration, paravertebral block) 12;*

*10 articles included in the systematic review and meta-analysis;*
