# Supplementary material for: Ultrasound-Guided Erector Spinae Plane Block in Thoracolumbar Spinal Surgery: A Systematic Review and Meta-Analysis
Source: Front Med (Lausanne). 2022 Jul 4;9:932101. doi: 10.3389/fmed.2022.932101 (PMC9289466; doi:10.3389/fmed.2022.932101)
Supplement: Supplementary file 3 [file Data_Sheet_3.docx]

| Reference | Was the study described as randomized (this includes words such as randomly, random, and randomization)? | Was the method used to generate the sequence of randomization described and appropriate (table of random numbers, computer-generated, etc)? | Was the study described as double blind? | Was the method of double blinding described and appropriate (identical placebo, active placebo, dummy, etc)? | Was there a description of withdrawals and dropouts? | Total score |  |
| --- | --- | --- | --- | --- | --- | --- | --- |
| Yörükoğlu et al. 2021 | 1 | 1 | 1 | 1 | 0 | 4 | |
| Yu et al. 2021 | 1 | 1 | 0 | 0 | 1 | 3 | |
| Zhang et al. 2020 | 1 | 1 | 1 | 1 | 1 | 5 | |
| Ciftci et al. 2020 | 1 | 1 | 0 | 0 | 0 | 2 | |
| Singh et al. 2019 | 1 | 1 | 0 | 0 | 1 | 3 | |
| Yayik et al. 2019 | 1 | 1 | 0 | 0 | 1 | 3 | |
| Goel et al. 2021 | 1 | 1 | 1 | 1 | 1 | 5 | |
| Finnerty et al. 2021 | 1 | 1 | 0 | 0 | 1 | 3 | |
| Yeşiltaş et al. 2021 | 1 | 1 | 1 | 1 | 1 | 5 | |
| Zhu et al. 2021 | 1 | 0 | 0 | 0 | 1 | 2 | |

**Assessment of methodological quality of included studies (Jadad scale).**

Low (<3), acceptable (3), good (4), and excellent (5).
